# Liaison nurse activities at hospital discharge: a strategy for continuity of care*

**DOI:** 10.1590/1518-8345.3069.3162

**Published:** 2019-08-19

**Authors:** Gisele Knop Aued, Elizabeth Bernardino, Judith Lapierre, Clémence Dallaire

**Affiliations:** 1 Faculdade de Santa Catarina, Florianópolis, SC, Brasil.; 2 Bolsista da Coordenação de Aperfeiçoamento de Pessoal de Nível Superior (CAPES), Brasil,; Programme des bourses des futurs leaders dans les Amériques 2016/2017, Canadá.; 3 Universidade Federal do Paraná, Curitiba, PR, Brasil.; 4 Université Laval, Faculté des Sciences Infirmières, Québec, QC, Canadá.

**Keywords:** Nursing, Continuity of Patient Care, Patient Discharge, Transitional Care, Professional Practice, Health Services Administration, Enfermagem, Continuidade da Assistência ao Paciente, Alta do Paciente, Cuidado Transicional, Prática Profissional, Administração dos Serviços de Saúde, Enfermería, Continuidad de la Atención al Paciente, Alta del Paciente, Cuidado de Transición, Práctica Profesional, Administración de los Servicios de Salud

## Abstract

**Objective:**

to describe the activities developed by the *liaison nurse*s for the continuity of care after hospital discharge.

**Method:**

descriptive, qualitative study, based on the theoretical reference. Strength Based Care. The sample comprised 23 *liaison nurse*s. The data was collected through a semi-structured questionnaire via Survey Monkey electronic platform and analyzed through the content analysis technique, with pre-defined categories.

**Results:**

among the liaison nurses, nine (39.14%), between 35 and 44 years of age; 17 (73.91%) were female; 15 (65.22%) were working eleven years or more nurse and 11 (47.82%), were between six and ten years old as a liaison nurse. The professionals participate in the identification of the patients who need care after hospital discharge, coordinate the planning of the hospital discharge and transfer the patient’s information to an extra-hospital service.

**Conclusion:**

the activities developed by the liaison nurses focus on the needs of the patient and the articulation with the extra-hospital services, and can be adapted to the Brazilian context as a strategy to minimize the discontinuity of care at the time of hospital discharge.

## Abstract (pt)

**Objetivo:**

descrever as atividades desenvolvidas pelas enfermeiras de ligação para a continuidade do cuidado após a alta hospitalar.

**Método:**

estudo descritivo, qualitativo, pautado no referencial teórico Cuidado Baseado nas Forças. A amostra compreendeu 23 enfermeiras de ligação. Os dados foram coletados por meio de um questionário semiestruturado via plataforma eletrônica *Survey Monkey* e analisados por meio da técnica de Análise de Conteúdo, com categorias pré-definidas.

**Resultados:**

entre as enfermeiras de ligação, nove (39,14%) possuíam de 35 a 44 anos de idade; 17 (73,91%) eram do sexo feminino; 15 (65,22%) trabalhavam há onze ou mais anos como enfermeira e 11 (47,82%) atuavam de seis a dez anos como enfermeira de ligação. As profissionais participam da identificação dos pacientes que necessitam de cuidados pós-alta hospitalar, coordenam o planejamento da alta hospitalar e transferem as informações do paciente para um serviço extra-hospitalar.

**Conclusão:**

as atividades desenvolvidas pelas enfermeiras de ligação centram-se nas necessidades do paciente e na articulação com os serviços extra-hospitalares e podem ser adaptadas ao contexto brasileiro como uma estratégia para minimizar a descontinuidade do cuidado por ocasião da alta hospitalar.

## Abstract (es)

**Objetivo:**

describir las actividades desarrolladas por las enfermeras de enlace para la continuidad del cuidado después del alta hospitalaria.

**Método:**

estudio descriptivo, cualitativo, pautado en el referencial teórico Cuidado Basado en las Fuerzas. La muestra comprendió 23 enfermeras de enlace. Los datos fueron recolectados por medio de un cuestionario semiestructurado vía plataforma electrónica *Survey Monkey* y analizados por medio de la técnica de Análisis de Contenido, con categorías predefinidas.

**Resultados:**

entre las enfermeras de enlace, nueve (39,14%), tenían 35 a 44 años de edad; 17 (73,91%), eran del sexo femenino; 15 (65,22%), trabajaban hace once o más años como enfermera y 11 (47,82%), actuaban de seis a diez años como enfermera de enlace. Las profesionales participan de la identificación de los pacientes que necesitan cuidados post-alta hospitalaria, coordinan la planificación del alta hospitalaria y transfieren las informaciones del paciente para un servicio extrahospitalario.

**Conclusión:**

las actividades desarrolladas por las enfermeras de enlace se centran en las necesidades del paciente y en la articulación con los servicios extrahospitalarios, y pueden ser adaptadas al contexto brasileño como una estrategia para minimizar la discontinuidad del cuidado con ocasión del alta hospitalaria.

## Introduction

Continuity of care is fundamental to the quality of health care, and is related to improved patient satisfaction, reduced costs, and decreased avoidable hospitalizations^1-3^. Continuity of care is a complex and multifaceted concept^2,4^. In this study, it is defined as the degree to which a series of events is experienced by the patient as coherent, connected and according to their needs^5^.

The combination of different elements results in continuity of care, such as: access to health services; good interpersonal skills; fluid information among professionals; appropriate coordination of care; integration of services^5^ and, above all, professional practices centered on the person, their needs and the available resources, whether those resources of the person or the health system.

In Latin America, continuity of care has been a challenge for health systems, because there is a lack of coordination between the different levels of care, resulting in difficulties in accessing health services, duplicity of diagnostic tests^6^, fragility regarding the articulation between the hospital and Primary Health Care (PHC) at hospital discharge, inefficiency or lack of counter-referral for patients with different health problems, incipient and ineffective hospital discharge planning^7-10^.

In order to find successful practices in the field of Nursing that effectively contribute to the continuity of care in the Brazilian context, a multi-centric project was developed in Canada, Spain and Portugal, focusing on the practices of nurses in the hospital discharge. These countries were chosen because they work with the liaison nurse, who has an important role in improving communication and coordination of care^11^. This study considers the results of the Canadian context.

A liaison person is a health professional designated to coordinate the discharge of the patient, to follow the care provided, and to transfer information from the hospital to the primary care professionals^12^. Liaison nurses are extremely important at hospital discharge to ensure that patients receive planned care according to their needs, regardless of where they will be assisted or the professionals who will assist them, and also, so that services of different levels of health care can operate as a network, in an articulated and coherent way.

A study on the general role of nurse liaison, regardless of the area of action, outlined six domains of practice of these professionals, being: care coordinator; educator; communicator; Advisor; lawyer of the patients; agent of change; contributor; negotiator; staff member and clinic, which pertains to the patient attending nurse based on a person-centered approach^11^.

With the person-centered approach, a relevant aspect of nursing liaison practice, this study was anchored in the theoretical framework of Strength Based Care, which argues that nurses need to learn new avenues to connect, engage, and initiate a movement that puts the patient in the center of care, with a focus on their uniqueness and their strengths^13^.

Strengths are the capacities that the person and the family have to face the challenges of life, facilitate their recovery, heal and collaborate for their well-being. Forces encompass a person’s attitudes, attributes, skills, resources, and abilities^13^. In addition, they are important social agents to unite the Nursing team in favor of care^14^.

It is argued that the knowledge of the activities developed by nursing nurses at hospital discharge can be useful to outline strategies for coping with the discontinuity of care in the Brazilian context. Thus, the question is: what are the activities developed by the nurses of connection for the continuity of care? The objective of this study was to describe the activities developed by the nurses of attachment for the continuity of care after hospital discharge.

## Method

A descriptive, qualitative study developed in the following hospital complexes in the province of Quebec: University Hospital Center of Montreal (UHCM) and University Hospital Center of Quebec (UHC). The participants were the liaison nurses who worked in these hospital complexes. No inclusion and exclusion criteria were established.

The recruitment of the participants took place with the express authorization of UHCM and UHC and was facilitated through the support of two Canadian researchers. Liaison nurses became aware of their research and goals through an informative meeting held in their work environment. Then, the heads of the liaison nurses sent one of the researchers the institutional email of the 36 liaison nurses. Subsequently, they were sent the invitation to participate in the survey via electronic platform Survey Monkey^®^.

At the invitation, the liaison nurses had the option of agreeing to participate in the survey or not. Upon agreeing to participate, the nurse was directed to the Survey Monkey platform with the immediate opening of the Free and Informed Consent Term (FICT). After reading the FICT and agreeing to such a document, by clicking on the yes option, the participant had access to the survey questionnaire. If it did not agree, clicking on the option did not automatically, the platform was closed.

Data collection was performed from March to July 2016 through a semi-structured questionnaire and preferably answered via Survey Monkey^®^ electronic platform or printed on paper, if the participant preferred. The paper questionnaire was a requirement of the Research Ethics Committee (REC) of partner institutions to respect nurses who did not feel comfortable using Survey Monkey^®^. For the liaison nurse who wished to respond to the questionnaire on paper, a copy of the questionnaire was sent via e-mail so that she could print, respond and then forward to one of the researchers by the email created specifically for this research.

After receiving the invitation, the deadline of 15 days for the participants to complete and send the questionnaire was established. For participants who did not respond within this time, an electronic reminder was sent again until the third attempt, with a 15 day interval. Of the 36 invitations sent, 24 were received within the established period, 23 of which were answered via the Survey Monkey^®^ platform and one via e-mail because it was answered on paper. Of the 24, one was deleted because it was incomplete. After exhaustive readings of the 23 questionnaires, the saturation of the data was perceived. Thus, the population was 36 and the sample totaled 23.

The semi-structured questionnaire was constructed based on the research objectives and literature on the subject. The questions sought to make explicit the characterization of the research participants, the identification of the patient who needs the liaison service, the planning of hospital discharge and its main elements and the transfer of patient information. The instrument was translated from Portuguese into French by two people who met the following criteria: being a nurse; have knowledge in the research topic and be fluent in Portuguese and French. Subsequently, the instrument was piloted by e-mail with two Canadian nurses who were not part of the research sample. After appropriate adjustments, the instrument was sent to a third nurse for a final pilot test.

Before starting the analysis, the data were translated from French into Portuguese by two fluent people in Portuguese and French, one of whom is a nurse and researcher. The analysis of the data was oriented by means of a matrix with pre-defined category of analysis. The categories of pre-defined analysis were: identification of the patient who needs the liaison service; hospital discharge planning; transfer of information between the hospital and other services.

The methodology used to analyze the data was Content Analysis, which consists of the set of techniques of the analysis of communications and comprises three stages: pre-analysis; exploitation of the material; data processing and interpretation. In the pre-analysis, the data was gathered in a Microsoft Word^®^ file and the floating readings were taken to know the text and allowed to invade the impressions and orientations. In the exploration of the material, codification and condensation of the recording units were carried out according to the pre-defined categories. Finally, the data were interpreted^15^through the theoretical reference Strength Based Care^13^.

In Brazil, the research project was approved by the REC of the Federal University of Paraná under the opinion n 1,426,575 and had as Certificate of Presentation for Ethical Assessment (CAAE) n 36975914.5.0000.0102. In Canada, it was approved by the REC of the participating institutions: at UHCM, under no. 888, 681, and at UHC, under no. 2015-2016-9012. The data collection took place after the approval of the RECs and the acceptance of the participants. To ensure anonymity, nurses were identified by the EL letters of the alphabet followed by a cardinal number in ascending order, according to the sequence in which the questionnaires were received.

## Results

Among nurses, nine (39.14%) were between 35 and 44 years of age, 17 (73.91%) were female, 15 (65.22%) worked eleven years or more as a nurse and 11 (47.82%) worked from six to ten years as liaison nurse. Next, the results of the research are presented according to the three pre-defined categories.

### Category 1: Identification of the patient who needs the liaison service

The identification of the patient who needs the liaison service can be performed by the liaison nurse, by the other professionals of the care team, and may also be intermediated by a member of the patient’s family.

When the nurse identifies the patient, she uses the active search, both individually and in partnership with nurses who occupy other positions in the hospital. Liaison nurses also identify patients during scheduled meetings with the multi-professional team. *It happens that I do active search for certain cases, for example, as soon as I make the lists of hospitalized users, every morning, I check if they are known or not […]* (EL14). *Active Search with the Chief Nurse Assistant or Nurse Responsible for Patient Care* (EL3). *[…] we also identified many patients at multidisciplinary meetings* (EL15).

When the identification is performed by a professional other than the liaison nurse, the liaison nurse informs the liaison service by sending a reference request via fax. *Physician, nurse practitioner, assistant nurse [...] physiotherapist, [...] social worker, nutritionist can identify and refer the patient to the liaison nurse* (EL7)*. […] the nurses send us a request via fax* (EL19).

In addition to hospital health care professionals, family members may also be involved in the process of identifying patients who need the liaison service. *The family can also make the request* (EL4).

### Category 2: Discharge Planning

Liaison nurses begin planning hospital discharge after identifying the patient who needs their services or after receiving the referral request, which can happen at different times of hospitalization and suffers interference from other variables, such as: clinical condition of the patient; completion of the documents by the health team; day of hospital discharge, not having a specific day to start planning for hospital discharge. *However, it is verified that, for the nurses of connection, the ideal is to initiate the planning of the hospital in the admission of the patient* (EL11). *Provided the medical prescriptions are in the medical record or by rehabilitation according to the physiotherapist and the occupational therapist* (EL20). *Very often, unfortunately, on the day of departure* (EL21).

However, it is verified that, for the nurses of connection, the ideal is to initiate the planning of the hospital in the admission of the patient. *Ideally, from their arrival* (EL22).

For the organization of the planning of the hospital discharge, the liaison nurses interview the patient and, if necessary, include a relative. During this interview, professionals evaluate the address and history of the patient. *Checking the address [...] other information usually found in the medical record (family doctor, history, medication list, allergies, reasons for admission)* (EL17).

Through the interview, the liaison nurses seek information about the patient’s home in order to verify if, after discharge, the patient can return to his or her home or if it is necessary to make some kind of adaptation. *Half life (residence for the elderly versus house / apartment) (EL20). Architectural barriers, adaptations* (EL23).

The need and availability of a person who can care for the patient and / or assist him / her in their daily living activities are also evaluated by the liaison nurses. This person can be a formal caregiver, a family member or a friend. *We evaluate [...] reference people who can help* (EL19).

A survey of out-of-hospital resources is performed by the liaison nurses, as they need to make sure that after the patients are discharged, the patients will receive the care according to their needs. Resources here refer to the presence of a primary care unit, a family doctor, a nurse who can continue the care received at the hospital, the availability of equipment, medications needed to treat the patient, among others. *Presence of equipment, resources, Local Health Service Center (LHSC), family physician, monitoring nurse* (EL4).

When planning hospital discharge, the nurses assess whether the patient and / or caregiver understood the guidelines provided to continue treatment. They also reinforce the care to be taken and the services available. *Verification of the education received for different care* (EL11). *[...] Disease management, X-ray management, blood glucose, etc.* (EL4). *Current knowledge about caregiving (EL8). Explanation about the care and services of the Local Health Service Center* (EL15).

To ensure high discharge planning according to the needs of the patient, the liaison nurses perform a concise physical examination, when necessary, and a psychic evaluation of the patient before hospital discharge *[…]in the interview, a summary physical assessment, for example a short-distance walking test to validate the safety of offsets* (EL14), *may occur. Assessment of remaining wounds and drains or other care* (EL21). *Their attitude towards return to the home, anxiety versus trust and his means* (EL18).

The patient’s family is included in the planning of the hospital discharge when it needs a person to perform the care or needs some adaptation at home, as well as at the request of the patient or when some family member shows interest in participating. *To the extent that the person is losing autonomy and that she needs support from her next* (EL2). *If home adjustment is required [...]* (EL7). *[...] when the family manifested the desire to be together in this process* (EL1). *At the request of the patient.* (EL5).

The main elements included in discharge planning and transferred to outpatient services depend on the situation of each patient and include socioeconomic data, health history, health conditions and care needs after hospital discharge. *Address [...]* (EL19). *The background* (EL23). *Major diagnoses* (EL1). *The care performed at the hospital* (EL1). *Latest laboratory results* (EL2). *Previous and current autonomy* (EL2). *Medicines in use* (EL1). *The care to be provided to the patient [...]* (EL6). *The way of life. The main helpers* (EL11).

### Category 3: Transfer of information between the hospital and other services

The liaison services of the hospital complexes have a computer system in which they share the patient’s information with an out-of-hospital service that, later, performs the necessary referrals. Thus, the transfer of information about the patient is performed by the liaison nurse, mostly, by sending the electronic form of the counter-referral to an extra-hospital service. *We have a computer system [...] it is sometimes direct or sometimes we send it via fax* (EL6).

The transfer of information between the hospital and the out-of-hospital service takes place at different times. For patients who require complex care, the information transfer occurs 24 to 48 hours before discharge. For patients in need of less complex care, information transfer occurs on the same day of hospital discharge. There are cases in which information transfer happens after discharge from the patient. *If the discharge is complex [...] 24 or 48 hours before discharge. If the request is simple, often the same day* (EL4). *[...] in certain stages are made when the patient has left* (EL8).

The transfer of patient information is reinforced by the delivery of some documents to patients at discharge, such as discharge prescriptions, discharge summary, information leaflets, among others, which can be delivered to primary care professionals or to other services for that they know what happened during the hospital stay and how they can continue the patient’s treatment. *Prescriptions, appointments marked, summary of hospitalization (EL2). Information brochures about your surgery, what to do […]* (EL6).

## Discussion

Liaison nurses actively participate in the process of identifying patients who need care after hospital discharge. In this process, it is fundamental that the nurses are open to dialogue with the patient, without judgments, because the patients and the family are predisposed to collaborate when they feel valued, understood, respected and safe^13^. The other professionals of the health team also identify the patients and refer them to the nurses, which demonstrates that all members of the health team have roles and responsibilities in the patient’s hospital discharge process^16^ and, consequently, with the continuity of care.

Among the forms of identification of the patients by the liaison nurses, these are highlighted by their role as coordinators of the hospital discharge process, since the liaison nurses are the points of convergence between the different members of the team and between the different health teams. In this context, communication is paramount for the liaison nurse to play her role as collaborative staff, which is key to maintaining patient-centered care^11^.

It is important to point out that the active search carried out by the nurses of liaison with the nurses who work in the care is an important strategy, since the nurse assistants are in direct contact with the patients, which allows them to make important observations about how the patients are responding to the patients. their health challenges^13^ and to identify the patients who really need care after hospital discharge.

Planning for hospital discharge is a process that needs to be started shortly after the patient is hospitalized, specifically within the first 24 hours. In this way, it is possible to identify the obstacles to discharge and to implement corrective actions^17^. The discharge planning, being a process, is characterized by different moments: in the admission, data can be collected related to the cognitive state, support systems and domestic environment; risk factors, such as the need for learning, can be evaluated near the discharge of the patient^16^.

The discharge planning does not only help the different health professionals to coordinate their services in a complementary way, but also to delineate a path of care expected for each patient, which promotes a sense of security to them and a basis for the taking shared decision^18^. In general, all inpatients require a discharge plan, which may be more or less specific^17^.

During the planning of hospital discharge, the liaison nurses investigate the strengths of each patient, which can be personal and external. The personal capacities of the patient, such as gait examination and laboratory tests without alterations, the ability to perform a particular care, the availability of a person who can assist the patient in his / her needs, patient’s trust attitude towards hospital discharge, and also the financial resources to make necessary adaptations at home are considered as examples of personal strengths.

External forces to the patient are present in the community, in the health system and include the availability of a health unit that has a nurse, family doctor and other professionals to provide patient care after hospital discharge and to provide the necessary equipment and medications to the treatment of the patient. Both the personal and external forces of the patients are fundamental to an effective continuity of care.

The use of the forces in the planning of the hospital discharge allows the nurses of connection have a holistic view of each patient, in that they make possible the evaluation of the physical, psychic, social conditions and of the environment in which it is inserted. Holism and indivisibility aim at integration, and this is only achieved when all aspects of the human being work in harmony. For this, nurses and other health professionals need to have a better knowledge of the patient and their families so that they can accompany them in their health and illness trajectory^13^.

For identification of strengths, the liaison nurse needs to look for them in the patient, in the family, and in the community; decide which are available and can be mobilized to deal with a specific problem or concern. What’s more, the bonding nurse can identify the potential forces that can be developed and the deficits that can turn into strengths, depending on the context of each patient^13^.

Different tools can be used during the discharge planning to better understand each patient. The genogram, a visual representation of family members, can be used to know about the family structure, its members and the relationship between them. The ecomapa, a graphic representation of the social network of the person that includes friends, health system, religious groups, among others, assists in the identification of available social support^13^.

Nurses, whose practice is based on Forces-Based Care, seek, in their patients and family, the skills that may be useful for recovery, development and survival. The attention of the nurse must be directed towards health, healing, alleviation of suffering, through actions that are inspired by external forces and resources, generating conditions that allow patients to achieve maximum functioning^13^.

In addition, nurses have the role of creating means to help the patient to become active in their learning process, because in each situation the patient needs to unravel their strengths and create new ones, such as developing certain dealing with the challenges that appear with an illness. Nurses should be aware of signs of readiness for learning, both of the patient and of the family members involved. When the patient is not ready for a particular experience, it is critical that the nurse provide support^13^.

The transfer of patient information between the hospital and other health services is established through the definition of an integrated computer system, which is in keeping with other studies that point to the need for a communication channel for the transfer of information between health services and professionals, such as: e-mail; telephone; systems and programs^11,19-20^.

Comprehensive care depends on an articulated health network so that patients’ problems can be treated at all levels of attention required for their solution and that access to these levels is appropriate and timely^21^. The use of a computer system that stores information about the patient and can be accessed independently of the level of attention the patient is being assisted in is fundamental, as there is no continuity of care without the sharing of quality information.

When there is no flow and mechanism defined for the transfer of information, many of these can be lost along the care network, which can lead to duplication in the actions of professionals and, consequently, increase in health costs, delay in solving problems and deficiency in the referral and counter-referral system. Therefore, it is fundamental that the transfer of the patient discharge planning information is coordinated and focused on a professional.

The counter-referencing is characterized as part of the competence of specialized attention and is presented as the mode of organization of the services configured in networks, supported by criteria, flows and mechanisms of agreement of operation, to guarantee the integral attention to the people through the facilitation access and continuity of care^22^.

One of the limitations of this study is not to include a description of the position of the nurses of liaison, which could contribute to a better discussion about the activities of the nurses of liaison. In addition, due to cultural, economic and social differences between Canada and Brazil, hospital institutions that want to implement the liaison position need to adapt certain activities according to the reality of each location.

As a breakthrough in the scientific field, it is worth highlighting the description of a set of activities that are little discussed in the scientific literature, which contributes to the dissemination of an important Nursing practice and that can be improved and adapted by nurse managers and by those who act directly on discharge from patients.

## Conclusion

The activities developed by Québec nursing nurses, at hospital discharge, point to a practice centered on the person and their family, with a view to ensuring the continuity of care to the patients, since in the process of hospital discharge they maintain a communication with their peers and other professionals, retrieve the patient’s history, identify the clinical and non-clinical needs of these patients, and act as educators and articulators between the services, transmitting information about the patient’s hospital discharge planning.

In view of the knowledge of the activities carried out by the liaison nurses, it is evident that it is important for hospital institutions to designate a professional to coordinate the patient’s hospital discharge process, acting as an articulator between the professionals, among the services of different levels of attention and advocating on behalf of the patient, since, without coordination actions, it is difficult to promote the continuity of care.

The role of the training centers for the position of liaison nurse is to spread the understanding that the patient is embedded in a health system, belongs to a family and to a community, and that each of these systems can, in different ways, contribute to patient recovery. The training centers also have the role of developing professionals who are able to work as a team, communicate effectively and be the link between services at different levels of care so that the health system operates in the form of a network.

The managers of the hospital institutions, who wish to implement the position of liaison nurse, need to take into account the experience of the nurse in the field of Nursing, her knowledge about the health system functioning and intra- and extra-hospital resources, the competence to recognize in the patient, in his family and in the health system the forces that contribute to improve the patient’s health conditions.

One of the contributions of this study to Nursing is the design of a strategy that effectively contributes to the advancement of continuity of care in the Brazilian context, through the implementation of the post of nurse liaison or of a liaison service in the hospital scope, since the activities of the liaison nurses can be transferred and adapted, depending on the context of each organization.

Another contribution to Nursing comes from the theoretical reference Strength Based Care because it is innovative, own of the Nursing, centered in the person, seeking the competences of the patients, of the families, the resources present in the health system and in the community, causing the nurse, initially reflect on the forces that are in favor of the patient and will help to solve the patients’ problems, rather than focusing on a list of problems.

In this study, the forces investigated by the nurses during the discharge planning involve the patient’s personal strengths, such as: favorable clinical conditions for their recovery; the availability of a person who can help the patient; the knowledge of the patient and the family about the care that should be performed; the patient’s confidence attitude towards hospital discharge and living conditions according to the needs. Likewise, external forces were investigated, such as the existence of health units for care after hospital discharge, the availability of nurses, family physicians, essential equipment and drugs for treatment.

The importance of the study, based on the theoretical reference Strength Based Care, is the development of a person-centered Nursing practice, in its potentialities and not only in its deficits, in its illness. The identification of the unique forces of each patient, community and health system is fundamental to promote the continuity of patient care.

For future research, it is suggested to study the profile of the patients attended by the nursing nurses as a way of knowing which patients are in need of the liaison service, as well as the impact of this service against certain indicators, such as: hospital readmission; patient satisfaction and patient perception of continuity of care.
